# Trait hierarchies are stronger than trait dissimilarities in structuring spatial co‐occurrence patterns of common tree species in a subtropical forest

**DOI:** 10.1002/ece3.7567

**Published:** 2021-05-01

**Authors:** Deyi Yin, Yu Liu, Qing Ye, Marc W. Cadotte, Fangliang He

**Affiliations:** ^1^ Key Laboratory of Vegetation Restoration and Management of Degraded Ecosystems, Guangdong Provincial Key Laboratory of Applied Botany South China Botanical Garden, Chinese Academy of Sciences Guangzhou China; ^2^ Center for Plant Ecology, Core Botanical Garden Chinese Academy of Sciences Guangzhou China; ^3^ Department of Biological Sciences University of Toronto‐Scarborough Toronto Ontario Canada; ^4^ ECNU‐Alberta Joint Lab for Biodiversity Study, Tiantong Forest Ecosystem National Observation and Research Station, School of Ecology and Environmental Sciences East China Normal University Shanghai China; ^5^ Ecology and Evolutionary Biology University of Toronto Toronto Ontario Canada; ^6^ Department of Renewable Resources University of Alberta Edmonton Alberta Canada

**Keywords:** functional traits, pairwise spatial association, spatial scale, trait dissimilarity, trait hierarchy

## Abstract

1. The dissimilarity and hierarchy of trait values that characterize niche and fitness differences, respectively, have been increasingly applied to infer mechanisms driving community assembly and to explain species co‐occurrence patterns. Here, we predict that limiting similarity should result in the spatial segregation of functionally similar species, while functionally similar species will be more likely to co‐occur either due to environmental filtering or due to competitive exclusion of inferior competitors (hereafter hierarchical competition).
2. We used a fully mapped 50‐ha subtropical forest plot in southern China to explore how pairwise spatial associations between saplings and between adult trees were influenced by trait dissimilarity and hierarchy in order to gain insight into assembly mechanisms. We assessed pairwise spatial associations using two summary statistics of spatial point patterns at different spatial scales and compared the effects of trait dissimilarity and trait hierarchy of different functional traits on the interspecific spatial associations. These comparisons allow us to disentangle the effects of limiting similarity, environmental filtering, and hierarchical competition on species co‐occurrence.
3. We found that trait dissimilarity was generally negatively related to interspecific spatial associations for both saplings and adult trees across spatial scales, meaning that species with similar trait values were more likely to co‐occur and thus supporting environmental filtering or hierarchical competition. We further found that trait hierarchy outweighed trait dissimilarity in structuring pairwise spatial associations, suggesting that hierarchical competition played a more important role in structuring our forest community than environmental filtering across life stages.
4. This study employed a novel method, by offering the integration of pairwise spatial association and trait dissimilarity as well as trait hierarchy, to disentangle the relative importance of multiple assembly mechanisms in structuring co‐occurrence patterns, especially the mechanisms of environmental filtering and hierarchical competition, which lead to indistinguishable co‐occurrence patterns. This study also reinforced the importance of trait hierarchy rather than trait dissimilarity in driving neighborhood competition.

## Graphical abstract

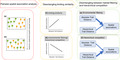

## 1 INTRODUCTION

The “entangled bank” metaphor of Darwin has inspired generations of community ecologists to explore the rules governing species co‐occurrence (Chesson, 2000; Gause, 1934; MacArthur, 1958; Ricklefs & Schluter, 1993; Storch et al., 2005; Tilman, 1982). Studies on species co‐occurrence in species‐rich communities over the past couple of decades have reinforced the importance of the relationship between trait‐mediated species differences and spatial distribution patterns among species for insights into the processes underlying patterns of biodiversity (Chesson, 2000, 2013; He & Biswas, 2019; HilleRisLambers et al., 2012; Kraft et al., 2015; Laughlin, 2014; Li et al., 2018).

Species differences, quantified by trait dissimilarity, are frequently used as a proxy for the niche differences among species that are believed to drive species co‐occurrence by influencing their response to environmental conditions and neighborhood interactions (Burns & Strauss, 2011; Cadotte et al., 2019; Cadotte & Tucker, 2017; Cavender‐Bares et al., 2009; Kraft & Ackerly, 2010). With such an approach, the environment is often assumed to act as a filter that selects for species possessing specific traits or trait values, leading to aggregated interspecific spatial associations between species with similar traits, while the pairwise spatial repulsion between species with similar traits is thought to result from limiting similarity via competition (Cavender‐Bares & Wilczek, 2003; He & Biswas, 2019). However, the assumed link between species differences and co‐occurrence only holds when the measured trait dissimilarity actually reflects niche differences and influences neighborhood competition (Cadotte et al., 2017). When these assumptions do not hold, for example, neighborhood competition is not driven by trait dissimilarity but by competitive advantage associated with particular trait values (i.e., trait hierarchy) (Carmona et al., 2019; Kraft et al., 2014; Kunstler et al., 2012), the pattern that species with similar functional traits co‐occur could also be the result of competitive exclusion of inferior competitors (hereafter hierarchical competition), not necessarily, or solely, due to environmental filtering (Cadotte & Tucker, 2017; Chesson, 2000; Lasky et al., 2014; Mayfield & Levine, 2010). Therefore, the relationship between interspecific spatial associations and species differences characterized by trait dissimilarity and trait hierarchy is key for disentangling the relative importance of multiple assembly mechanisms, especially those leading to similar co‐occurrence patterns, for example, environmental filtering and hierarchical competition.

**FIGURE 1:**
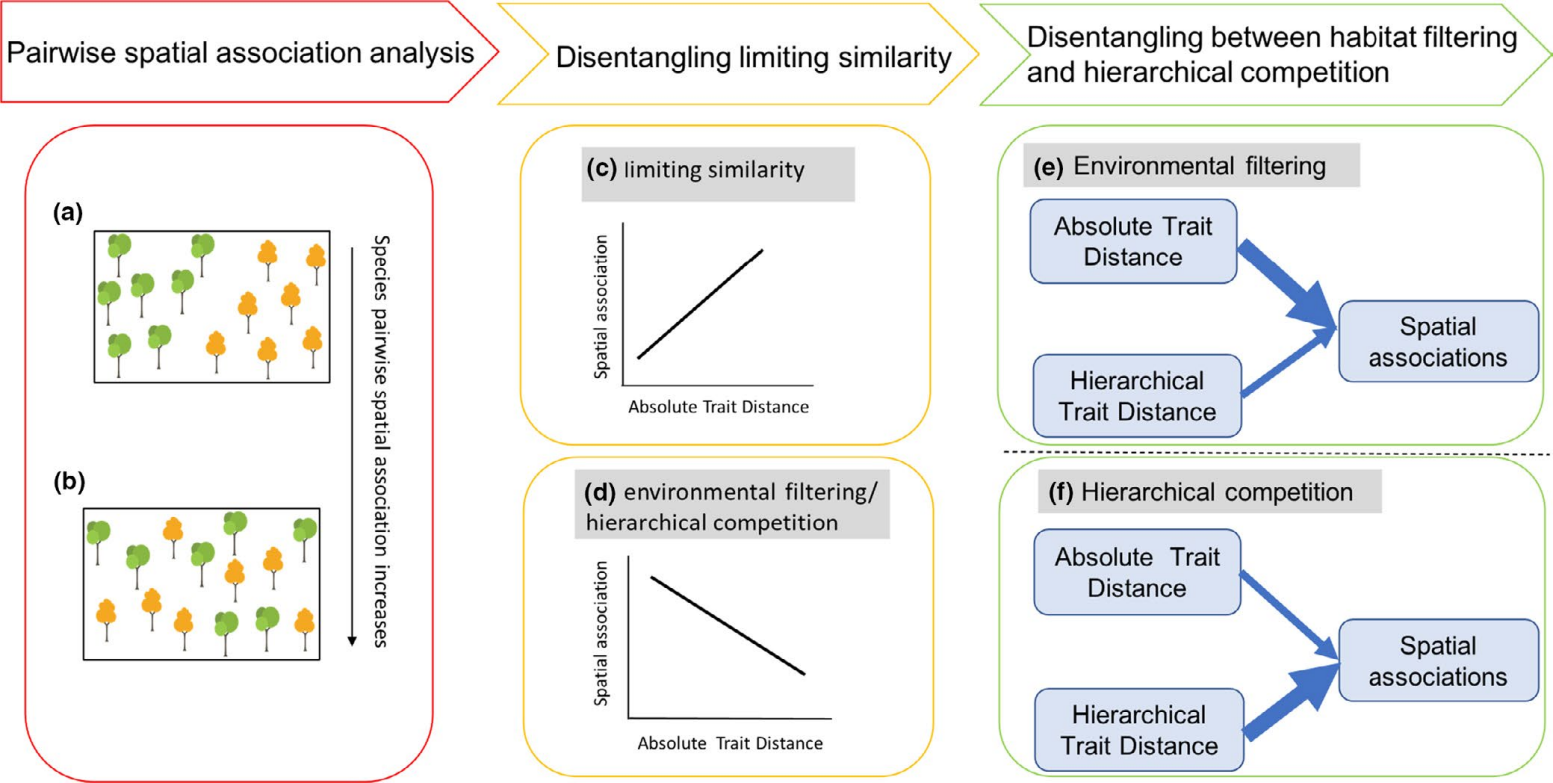
Conceptual framework to illustrate hypotheses of this study. (a) and (b), respectively, show spatial associations between repulsion and attraction between two species at coarse spatial scale. (c) and (d) show the predicted relationships between pairwise spatial associations and absolute trait distance under different processes of community assembly: (c) limiting similarity, if absolute trait distance has positive effects on pairwise spatial associations; and (d) environmental filtering or hierarchical competition, if absolute trait distance has negative effects on pairwise spatial associations. In the case of (d), if absolute trait distance has stronger effects on pairwise spatial association than hierarchical trait distance, we infer that environmental filtering mainly drives the co‐occurrence pattern (e); if the hierarchical trait distance has stronger effects on pairwise spatial associations than absolute trait distance, the effect of hierarchical competition is thought to drive the co‐occurrence pattern (f)

Trait dissimilarity and trait hierarchy can be characterized, respectively, as absolute (i.e., nondirectional) and hierarchical (i.e., directional) interspecific trait differences and can, to a certain extent, serve as an indirect measure of species niche differences (especially by the multiple trait dissimilarity) and fitness differences (Carmona et al., 2019; Kraft et al., 2014, 2015; Kunstler et al., 2012). Bivariate spatial point pattern analysis is a primary tool for estimating the degree of segregated or aggregated pairwise species co‐occurrence patterns (Figure 1a,b), and understanding the underlying processes that create these nonrandom patterns (He & Duncan, 2000; Wiegand et al., 2007; Wiegand & Moloney, 2014). Associations with trait dissimilarity and trait hierarchy provide the bivariate analysis with a basis for detecting the relative importance of multiple assembly processes (Carmona et al., 2019; Kunstler et al., 2012, 2016; Lasky et al., 2014; Shen et al., 2013; Wiegand et al., 2007, 2017).

Beyond this logic, the relative importance of different assembly mechanisms and their signatures on spatial associations is highly scale‐dependent (Gianuca et al., 2016; Smith et al., 2013; Zhang et al., 2020) and might vary with plant life stages (Spasojevic et al., 2014). For example, plants are most likely to interact with their adjacent neighbors (e.g., within a few meters), while environmental filtering often occurs beyond neighborhood scales (e.g., from tens to hundreds of meters), revealing different biodiversity patterns and assembly mechanisms across spatial scales (Jin et al., 2020; Wiegand et al., 2017). As for ontogeny, saplings are more susceptible to biotic interactions, while abiotic filtering might be more important among co‐occurring adults (Spasojevic et al., 2014). Therefore, the spatial pattern–trait difference relationships might shift in the relative importance of different assembly mechanisms across spatial scales and during ontogeny.

In this study, we predict that limiting similarity should result in functionally similar species occupying segregated areas, leading to a positive relationship between the absolute functional trait distance (trait dissimilarity) and pairwise spatial associations (Figure 1c). Conversely, functionally similar species are expected to co‐occur if environmental filtering or hierarchical competition dominates (HilleRisLambers et al., 2012; Mayfield & Levine, 2010), leading to a negative relationship between the absolute functional trait distance and spatial association (Figure 1d). To further disentangle which of environmental filtering and hierarchical competition is responsible for the pattern of co‐occurrence of functionally similar species, it is necessary to simultaneously test and compare the relative strengths of trait dissimilarity and trait hierarchy on pairwise spatial associations. If environmental filtering prevails, we expect that the strength of trait dissimilarity should be greater than that of trait hierarchy (Figure 1e), and if hierarchical competition drives community patterns, the effects of trait hierarchy are expected to be stronger than that of trait dissimilarity (Figure 1f).

To link forest assembly mechanisms to spatial pattern–trait difference relationships and test the three hypotheses above (i.e., limiting similarity, environmental filtering, and hierarchical competition), we addressed the following questions about spatial associations: (1) How are pairwise spatial associations related to trait dissimilarity and trait hierarchy? (2) Do the spatial pattern–trait difference relationships remain consistent across life stages (i.e., sapling vs. adult trees) and (3) across different spatial scales? To address these questions, we firstly analyzed the bivariate spatial associations of tree species across two life stages (i.e., sapling and adult trees) at three different spatial scales, that is, local (*r* = 5 m), intermediate (*r* = 30 m), and large (*r* = 50 m) scales in a fully mapped 50‐ha (1,000 × 500 m) plot in the Heishiding Nature Reserve in southern China using spatial point pattern analysis. To reveal how trait dissimilarity and hierarchy determine species co‐occurrence patterns in the study forest, we then evaluated the support for the three hypotheses by assessing and comparing how trait dissimilarity and hierarchy determine species co‐occurrence patterns in the study forest across life stages and different spatial scales.

## 2 MATERIALS AND METHODS

### 2.1 Dataset

The study area is located in the Heishiding Nature Reserve (HSD; 111°52 E, 23°27N), Guangdong Province, China. Stems with diameters at breath height (DBH) ≥ 1 cm were measured, identified, and mapped in a 50‐ha plot established in 2013, providing us with the distribution and abundance of 213 tree/shrub species with 213,969 individuals in total (Yin & He, 2014). The HSD plot is one of the sites of the CTFS‐Forest Global Earth Observatory, which is a worldwide network dedicated to advancing long‐term study of the world's forests (http://www.ctfs.si.edu; Anderson‐Teixeira et al., 2015).

We chose saplings (with DBH between 1 and 3 cm) and adult trees (with DBH >10 cm) for analysis in this study. To obtain a sufficiently large sample size for point pattern analyses, we only included common tree species that have at least 50 individuals each species at each selected DBH level for analysis. In total, we had 137 species for saplings with 119,074 individuals (accounting for 66.5% and 99.2% of the number of species and individuals for saplings in the forest, respectively) and 80 adult tree species with of 27,453 individuals (accounting for 54.4% and 88.8% of the number of species and individuals for adult trees in the forest, respectively) in this analysis, together accounting for 68.5% of the total individuals in the forest.

### 2.2 Spatial point pattern analysis of pairwise species association

We test the null hypothesis that species pairs are spatially independent, as opposed to patterns of repulsion or attraction. If two species show segregation in their spatial distributions, we will find fewer points of species *j* within the neighborhood of species *i* than expected under independence of the two species. Conversely, if the two species show attraction in their spatial distributions, we will find more points of species *j* within the neighborhood of species *i* than expected. To assess pairwise spatial associations, we used seminal techniques of bivariate point pattern analysis based on the distributions of distances of all pairs of points between the two species (Lotwick & Silverman, 1982; Wiegand & Moloney, 2014; Wiegand et al., 2017). Two summary statistics, bivariate pair correlation function (pcf) *g_ij_*(*r*) and bivariate distribution function *D_ij_*(*r*) of nearest neighbor distances, were used in this analysis. The bivariate pair correlation function *g_ij_*(*r*) can be estimated using the quantity *λ_j_g_ij_*(*r*), where *λ_j_* is intensity (i.e., density) of species *j* in the whole study area, measuring the mean density of trees of species *j* at distance *r* away from a tree of the focal species *i* (Ripley, 1981; Stoyan & Stoyan, 1994). *D_ij_*(*r*) could be defined as the probability that trees of the focal species *i* have their nearest species *j* neighbor(s) within distance *r* (Diggle, 1983). *D_ij_*(*r*) can provide additional information of the spatial patterns that is not provided by the bivariate pair correlation function *g_ij_*(*r*), especially in the extremely heterogeneous cases for focal species, for example, many individuals of focal species *i* have no species *j* neighbor but few have many species *j* neighbors (Wang et al., 2010; Wiegand et al., 2007).

The independence of bivariate spatial point patterns is examined through the comparison of the summary statistics of the observed bivariate patterns with those of the null model, that is, the observed patterns are compared against the simulated null model to test whether the hypothesis holds. In this study, we implemented the null model by keeping the locations of the focal species *i* unchanged while randomizing the distribution of species *j* by the method of toroidal shift, which maintains most of structure of species *j* (Lotwick & Silverman, 1982). The null model of toroidal shift removes the effects of environmental heterogeneity and the interspecific interactions, while retains the spatial structures of individual species. If a summary statistic of the observed bivariate spatial pattern significantly differs from the expectation of the null model, it is reasonable to conclude that the departure results from species interactions or environmental heterogeneity.

To assess the magnitude of departures from the null model, for each species pair and for each observed summary statistic *S*_0_(*r*) (i.e., *g_ij_*(*r*) or *D_ij_*(*r*)), we computed their standardized effect size *z*(*r*) as follows:

(1) $$z\left(r\right)=\frac{S_{0}\left(r\right)‐\mu_{\text{null}}\left(r\right)}{\sigma_{\text{null}}\left(r\right)},$$

where *S*_0_(*r*) is the observed summary function (either *g_ij_*(*r*) or *D_ij_*(*r*)), and *μ*_null_(*r*) and *σ*_null_(*r*) are, respectively, the average and the standard deviation of the summary functions for 999 bivariate patterns simulated according to the null models (Chanthorn et al., 2018; Wang et al., 2018; Wiegand et al., 2016). For a given distance *r*, the hypothesis of independence for a species pair can then be accepted if −*z_α_*(*r*) < *z*(*r*) < *z_α_*(*r*) at a given pointwise significance level of *α*. For *α* = .05, *z_α_* = 1.96, which is equivalent to testing whether the observed summary statistic is located within the 2.5th and 97.5th percentiles of the corresponding null model distribution. When *z*(*r*) > 1.96, the observed summary statistic is larger than the expectation of the null model with error rate *α* = .025, and the species pairs are spatially attracted at distance *r*. While *z*(*r*) < −1.96 suggests repulsion at distance *r*. The distance *r* in this study was chosen to be 5, 30, and 50 m to test the effect of scale on spatial patterns. Because the association between two species might be asymmetric, we analyzed the spatial patterns between two species twice with each species serving as the focal species, that is, species *i* versus species *j* and species *j* versus species *i*. Specifically, we examined the interspecific spatial associations of 137 × 136 = 18,632 species pairs for saplings and 80 × 79 = 6,320 species pairs for adult trees in this study for two different summary statistics of bivariate spatial point pattern analysis: *g_ij_*(*r*) and *D_ij_*(*r*). All the spatial association analyses were conducted in R (R Core Team, 2020) and using the package of “spatstat” (Version 1.62‐2, Baddeley et al., 2015).

### 2.3 Species trait dissimilarity and hierarchy

We focused on six key functional traits here: leaf area (LA; cm^2^), specific leaf area (SLA; cm^2^/g, calculated as leaf area/dry mass), leaf dry matter content (LDMC; g/g, calculated as leaf dry mass/fresh mass), wood density (WD; g/cm^3^, calculated as trunk wood dry mass/fresh volume), wood dry matter content (WDMC; g/g, calculated as dry wood mass/fresh wood mass), and tree maximum height (*H*_max_; m) for each of the selected species in this study. These traits represent leading axes of ecological variation among tree species that have been previously implicated in interspecific variation in resource use efficiency, species interactions, and life history strategies and are frequently used in analyses of the functional structure of forest communities (Kraft & Ackerly, 2010; Kunstler et al., 2016; Li et al., 2018). Specifically, LA is important for energy balance and hydraulic architecture (Ackerly & Cornwell, 2007). SLA is a key element of the leaf economic spectrum and correlates with procurement of resources (Wright et al., 2004). LDMC is indicative of a plant species’ resource use strategy that links to the trade‐off between a rapid assimilation and growth (Díaz et al., 2004). WD is significant in relation to growth, stress tolerance, and survival rates (Chave et al., 2006), and WDMC is related to wood defense and persistence (Costa et al., 2018; van der Sande et al., 2018). *H*_max_ is a key determinant of light competition (Westoby et al., 2002). Data of leaf traits (LA, SLA, and LDMC) were randomly collected and measured from 30 individuals for each common tree species in the HSD plot (He et al., 2018), while the trunk wood core from 5–20 individuals for each species was extracted at a height of 1.3 m to measure the wood traits (WD and WDMC) (He & Deane, 2016). For *H*_max,_ we calculated it for each species by computing the 99% quantile of the height measurements in the plot.

We calculated two kinds of species differences based on each individual trait: absolute trait distance and hierarchical trait distance, to evaluate the effects of trait dissimilarity and trait hierarchy on interspecific spatial associations, respectively (Carmona et al., 2019; Kraft et al., 2014; Kunstler et al., 2012). Absolute trait distance between species *i* and species *j* was calculated as |*t_i_*‐*t_j_*|, where *t_i_* and *t_j_* are the functional trait values of the respective species, while hierarchical trait distance was calculated as *t_i_*‐*t_j_*. In both trait distance measures, species *i* is the focal species in correspondence with that in the pairwise spatial point pattern analysis. As species’ niche dissimilarity might be better measured by a multitrait than by a single‐ trait approach (Kraft et al., 2015), we used species scores along the first axis of a principal component analysis of the above six trait data (accounting for 44% and 45% of the variation among species of saplings and adult trees, respectively) as an integrated trait measurement to calculate both absolute and hierarchical trait distances (hereafter PCA) (Kraft et al., 2014). All trait differences, including trait absolute and hierarchical distances of each individual trait and the integrated trait PCA, were centered and standardized to facilitate comparison in the subsequent analyses.

### 2.4 Relationships between pairwise spatial associations and trait dissimilarity and hierarchy

The pairwise spatial associations (estimated as SES of *g_ij_*(*r*) and *D_ij_*(*r*), represented as *z_ij_* below) were modeled as a function of trait dissimilarity and trait hierarchy between species *i* and species *j*, in a linear mixed model using the “lmer” in the R package “lme4” (Bates et al., 2015), in which the focal species were treated as random intercept allowing intercepts to vary among each focal species, and we used each explanatory predictor as random slopes to evaluate the effects of each predictor on spatial associations for different focal species. The model takes the general form:

(2) $$z_{\mathrm{ij}}=a+a_{i}+\left(b+b_{i}\right){\text{pred}}_{\mathrm{ij}}+\varepsilon_{\mathrm{ij}},$$

where *a* is the fixed intercept and *b* is the fixed coefficient of the explanatory predictor for the regression, while *a_i_* is the random intercept and *b_i_* is the random coefficient for the explanatory predictor for the focal species *i*. *z_ij_* represents the spatial associations between species *i* and species *j* with the focal species *i*, and pred*_ij_* represents the explanatory predictor of trait distance, which could either be absolute or hierarchical trait distances between species *i* and species *j*. Pairwise spatial associations (*z_ij_*), which were measured by two different summary statistics (i.e., *g_ij_*(*r*) and *D_ij_*(*r*)) across three spatial scales (i.e., 5, 30, and 50 m), were modeled as a function of either the absolute or the hierarchical trait distance of LA, SLA, LDMC, WD, WDMC, Hmax, and the integrated trait PCA, separately.

We first exclusively applied the absolute trait distances of six individual traits: LA, SLA, LDMC, WD, WDMC, and *H*_max_ and the integrated trait PCA to model in Equation (2) to evaluate the effects of absolute trait distances on the pairwise spatial associations to distinguish the assembly mechanisms of limiting similarity (Figure 1c) and environmental filtering or hierarchical completion (Figure 1d). If absolute trait distances have positive effects on pairwise spatial associations (positive coefficient *b* in Equation 2), it suggests functionally similar species tend to be spatially repulsive and indicates the operation of limiting similarity via competition in the forest (Figure 1c). If absolute trait distances have negative effects on pairwise spatial associations (negative coefficient *b* in Equation 2), it indicates functionally similar species tend to co‐occur, presumably caused by either environmental filtering or hierarchical competition (Figure 1d) that needs to be further tested.

To further test the mechanisms of environmental filtering and hierarchical competition when absolute trait distances have negative effects on pairwise spatial associations (Figure 1d), we applied predictor variables of both absolute and hierarchical trait distances of the six individual functional traits and the integrated trait PCA to model (2), separately, and compared the coefficients of the predictor variables of absolute trait distances and their corresponding hierarchical trait distances for each focal species to assess the relative importance of absolute and hierarchical trait distances of each trait in explaining the pairwise spatial associations. To do that, we plotted and compared the distributions of absolute values of the coefficients of the predictor variables estimated from model (2) for each focal species for each trait.

## 3 RESULTS

### 3.1 Pairwise spatial associations

**FIGURE 2:**
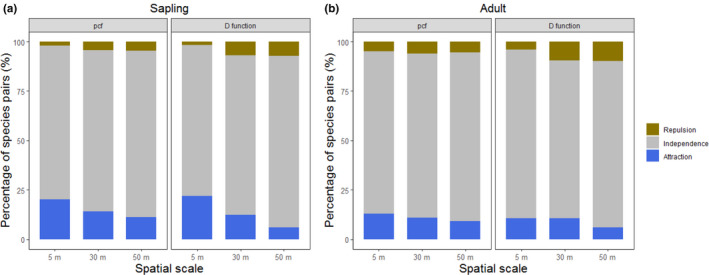
The percentages of different types of pairwise spatial point patterns assessed by the standardized effect size (SES) of two different summary statistics, bivariate pair correlation function (*g_ij_*(*r*), pcf), and bivariate distribution function of nearest neighbor (*D_ij_*(*r*), D function), at three spatial scales (*r* = 5, 30, and 50 m) for saplings (the left panel (a)) and adult trees (the right panel (b))

The pairwise spatial associations assessed by *g_ij_*(*r*) and *D_ij_*(*r*) indicate that interspecific spatial independence (−1.96 ≤ *z*(*r*) ≤ 1.96) was the dominant pattern across the three different spatial scales, accounting for around 80% of the total number of species pairs for both saplings and adult trees (Figure 2). The pattern of repulsion accounts for a small proportion of species pairs (1.8%–7.2% for saplings and 4.0%–9.8% for adult trees, respectively). Attraction was more commonly observed (accounting for 6.0%–22.0% for saplings and 6.0%–12.9% for adult trees, respectively) than repulsion across spatial scales for both spatial summary statistics (Figure 2). We also noticed that there was a trend that the proportion of repulsive species pairs increased with spatial scales, while the proportion of attractive species pairs decreased with spatial scales (Figure 2).

### 3.2 Relationships between spatial pattern and absolute trait distances

**FIGURE 3:**
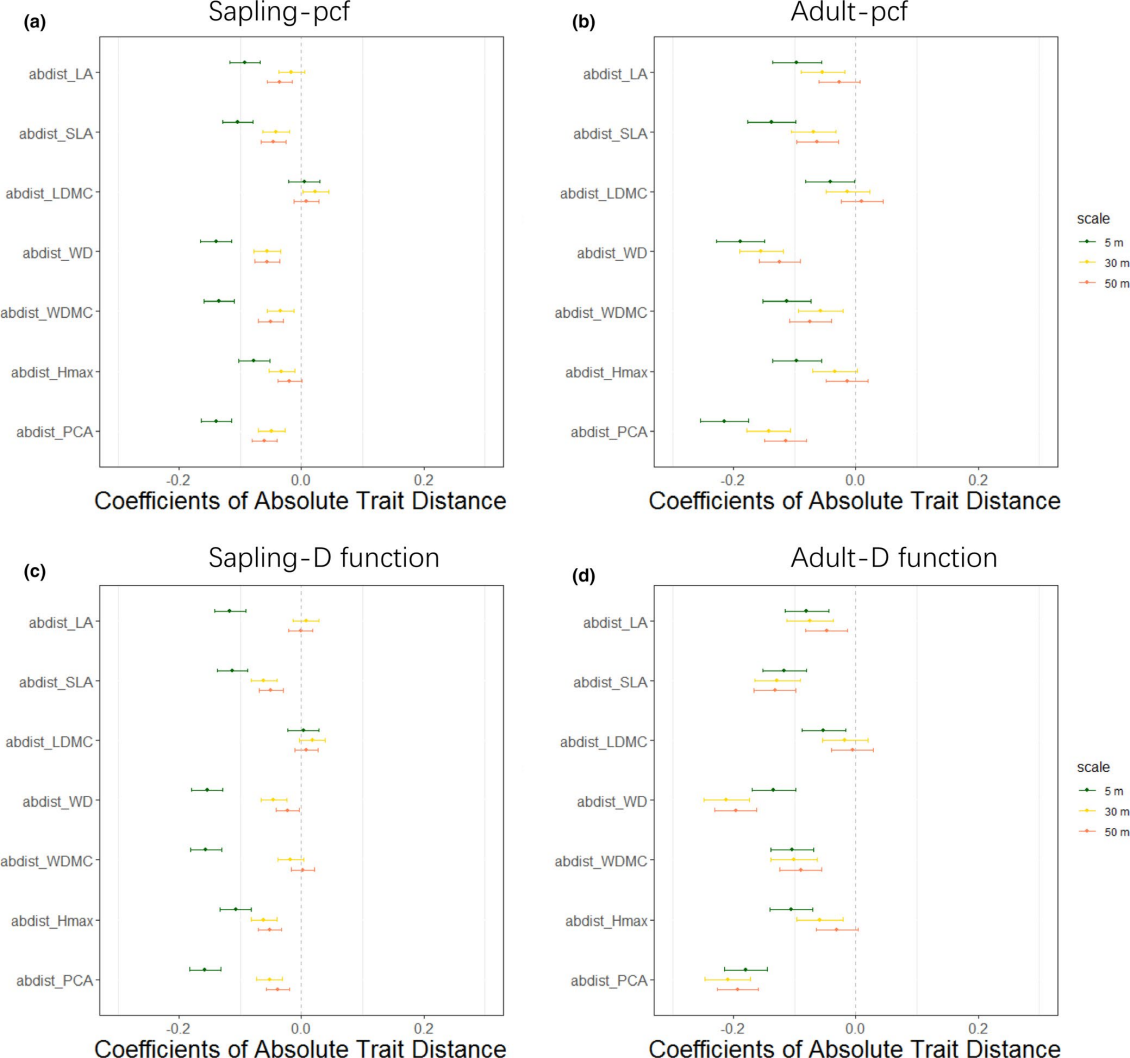
Effects of absolute trait distances on the pairwise spatial associations in Equation (2) that only includes the absolute trait distances of individual traits or integrated trait PCA as explanatory predictors. The left panels (a) and (c) show the effects of absolute trait distances on pairwise spatial associations assessed by bivariate pair correlation function (*g_ij_*(*r*), pcf) and bivariate distribution function of nearest neighbor (*D_ij_*(*r*)), respectively, for saplings across different spatial scales at *r* = 5, 30, and 50 m. The right panels (b) and (d) show the effects of absolute trait distances on pairwise spatial associations assessed by bivariate pair correlation function (*g_ij_*(*r*), pcf) and bivariate distribution function of nearest neighbor (*D_ij_*(*r*)), respectively, for adult trees across different spatial scales at *r* = 5 m, 30 m, and 50 m. *H*_max_, maximum height; LA, leaf area; LDMC, leaf dry matter content; SLA, specific leaf area; WD, wood density; WDMC, wood dry matter content. PCA means the integrated trait values calculated as scores along the first axis of a principal component analysis of the above six trait data, and abdist means absolute trait distance

By fitting the linear mixed regression model (2) using absolute trait distances in individual traits exclusively, we found statistical support for negative effects of the absolute trait distances measured by individual and integrated traits on pairwise spatial associations assessed by the two summary statistics (*g_ij_*(*r*) and *D_ij_*(*r*)) across different spatial scales for both saplings and adult trees (Figure 3). While only the absolute trait distance of LDMC did not show significant effects on pairwise spatial associations for both saplings and adult trees across spatial scales for the two summary statistics (Figure 3), the fixed coefficients of absolute trait distances of individual traits of SLA, WD, and the integrated trait PCA were consistently negative for both summary statistics across life stages and spatial scales. The absolute trait distances of LA, WDMC, and *H*_max_, in general, also had negative effects on pairwise spatial associations, with the exception that the effects of absolute trait distance of LA and WDMC on pairwise spatial associations of saplings assessed by *D* function at intermediate and large spatial scales (*r* = 30 m and 50 m), and the effects of absolute trait distance of *H*_max_ on adult spatial associations assessed by pcf at *r* = 50 m, were not significant. It is noteworthy that in general the strength of absolute trait distances on spatial associations decreased with spatial scales (Figure 3).

### 3.3 Comparison between the strengths of trait dissimilarity and hierarchy on spatial patterns

**FIGURE 4:**
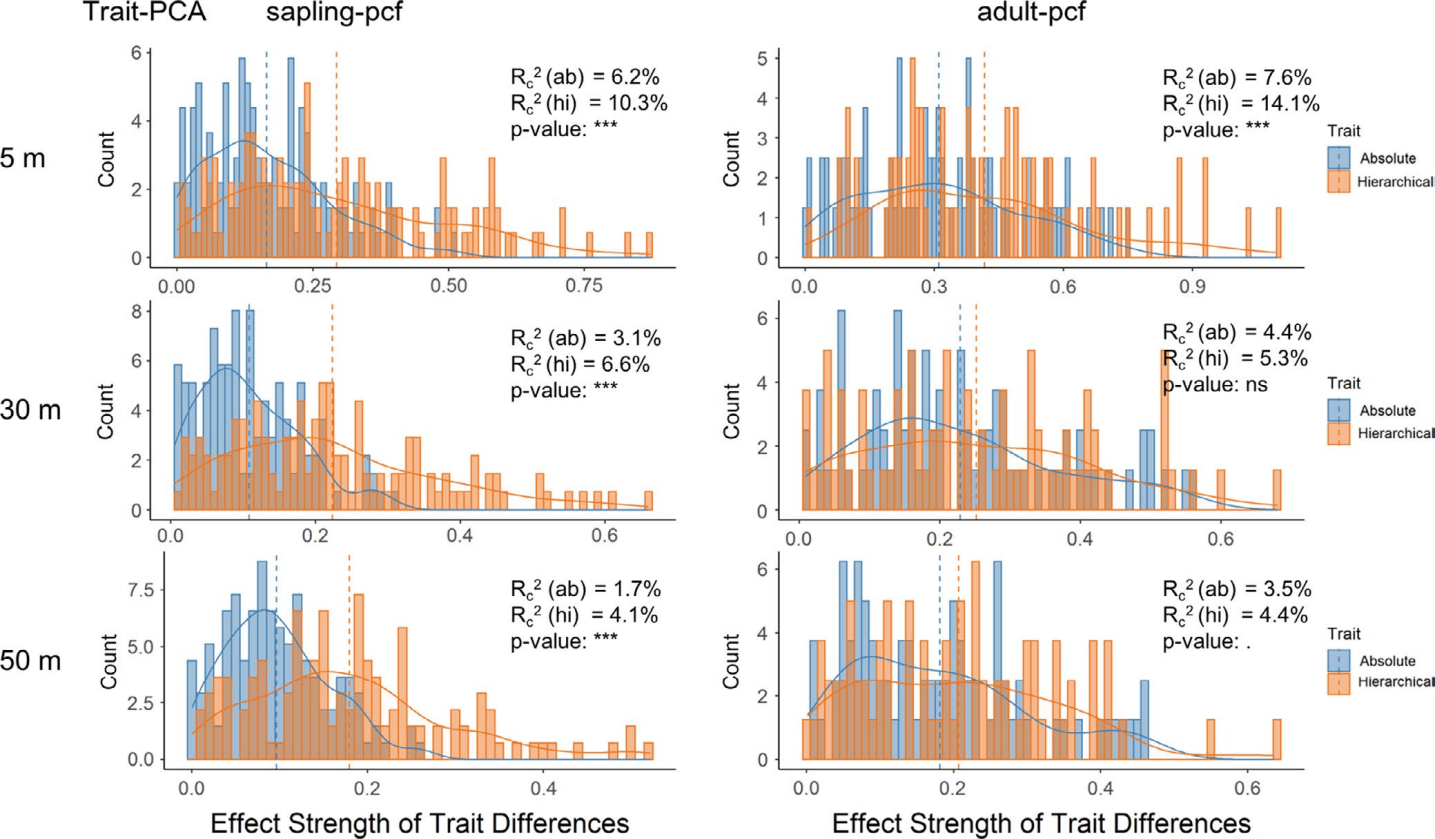
Comparison between the strengths of absolute and hierarchical trait distances of the integrated trait PCA on spatial associations for the foal species of sapling and adult trees. The strengths of absolute and hierarchical trait distances were, respectively, given by the absolute values of the coefficients of the variables of hierarchical and absolute trait distances of different functional traits in the model of Equation (2). Histograms, distributions, and mean values of absolute values of the coefficient estimated for each focal species are plotted (blue for absolute trait distance and orange for hierarchical trait distance). The conditional *R*‐squared (*R*_c_^2^) for each model and p‐value for the paired *t* test for the strengths of absolute and hierarchical trait distances for each focal species are reported in each panel, where *** indicates *p* < .001, ** indicates *p* < .01, * indicates *p* < .05, . indicates *p* < .1, and ns indicates *p* > .1. The results presented here are for spatial associations assessed by bivariate pair correlation function (*g_ij_*(*r*), pcf) at *r* = 5, 30, and 50 m

**FIGURE 5:**
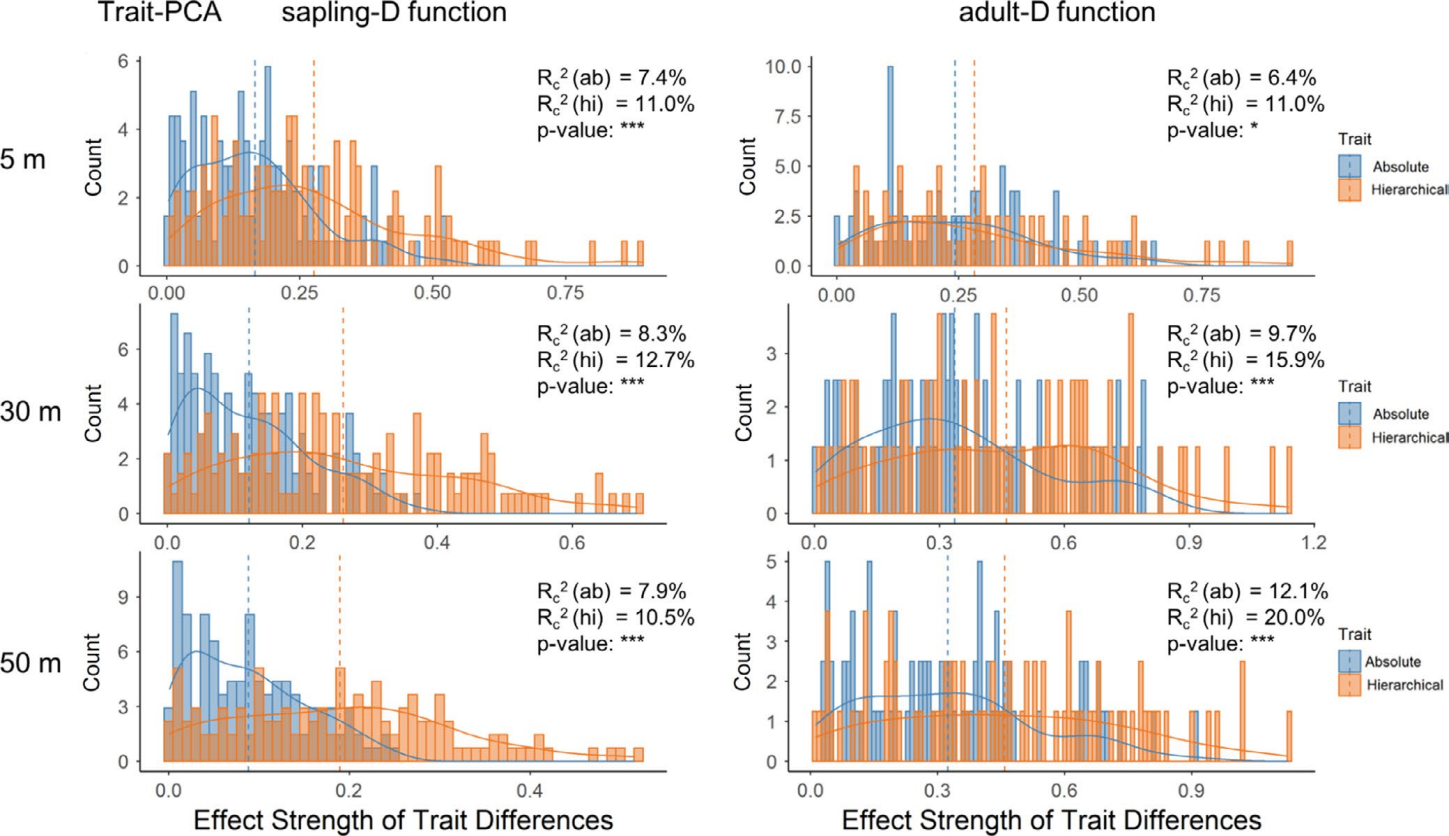
Comparison between the strengths of absolute and hierarchical distances of the integrated trait PCA on spatial associations for the foal species of sapling and adult trees. The strengths of absolute and hierarchical trait distances are, respectively, given by the absolute values of the coefficients of the variables of hierarchical and absolute trait distances of different functional traits in the model of Equation (2). Histograms and density distributions of absolute values of the coefficient estimated for each focal species are plotted (blue for absolute trait distance and orange for hierarchical trait distance). The conditional *R*‐squared *R*_c_^2^ for each model and *p*‐value for the paired *t* test for the strengths of absolute and hierarchical trait distances each focal species are reported in each panel, where *** indicates *p* < .001, ** indicates *p* < .01, * indicates *p* < .05, . indicates *p* < .1, and ns indicates nonsignificant. The results presented here are for spatial associations assessed by bivariate distribution function of nearest neighbor (*D_ij_*(*r*), D function), at *r* = 5, 30, and 50 m

Results in Figure 3 show that the absolute trait distances of individual and integrated traits generally had negative effects on pairwise spatial associations, which indicates the absence of limiting similarity and supports environmental filtering or hierarchical competition (Figure 1d). We thus compared the strengths of both trait hierarchy and trait dissimilarity on pairwise spatial associations using model (2) to distinguish these two mechanisms. For both saplings and adult trees, we found that LA (Figures S1 and S2), SLA (Figures S3 and S4), WD (Figures S5 and S6), and WDMC (Figures S7 and S8) consistently showed stronger trait hierarchy effects on pairwise spatial associations than trait dissimilarity effects across different summary statistics and spatial scales. For the integrated trait PCA shown in Figures 4 and 5, we found that the strength of trait hierarchy on pairwise spatial associations was consistently stronger than the strength of trait dissimilarity for saplings and for most cases of adult trees, with the exception of spatial associations assessed by pcf at *r* = 30 m, where trait dissimilarity and hierarchy showed no significant differences. For *H*_max_ (Figures S9 and S10), the strength of trait hierarchy was also consistently stronger than the strength of trait dissimilarity effects for saplings. However, for adult trees, trait dissimilarity of *H*_max_ showed stronger (e.g., pcf at *r* = 30 and 50 m) or comparable (e.g., pcf at *r* = 5 m and D function at *r* = 5, 30 and 50 m) effects on spatial associations than trait hierarchy. The trait dissimilarity of LDMC did not show significant effects on spatial associations (Figure 3), suggesting LDMC did not contribute to limiting similarity, environmental filtering, or hierarchical competition. We therefore did not present the comparison between the strengths of trait dissimilarity and hierarchy of LDMC on spatial patterns.

## 4 DISCUSSION

Trait dissimilarity effects were widely considered to explain species co‐occurrence over the past decade (Burns & Strauss, 2011; He & Biswas, 2019; Kraft & Ackerly, 2010). Consistent with the findings of He and Biswas (2019), we observed negative relationships between trait dissimilarity and pairwise spatial associations in this study across plant life stages, summary statistics, and spatial scales for individual and integrated functional traits, except LDMC that showed nonsignificant effects on pairwise spatial associations (Figure 3). However, instead of simply interpreting this negative relationship as a result of environmental filtering and an absence of competition as reported in He and Biswas (2019), we provided support for the hypothesis that the effects of hierarchical competition on the co‐occurrence pattern, which could produce a pattern indistinguishable from that expected under environmental filtering, were greater than the effects of environmental filtering in the study forest (Figures 4 and 5, Figures S1–S10).

The negative relationship between trait dissimilarity and pairwise spatial associations was typically interpreted as evidence for the relative unimportance of competition and instead supporting the inference that assemblages were structured by environmental filtering (He & Biswas, 2019). However, this interpretation could be misleading because the negative relationship between trait dissimilarity and pairwise spatial associations could also be caused by neighborhood competition that selects species with particular trait values independent of environmental filtering (Carmona et al., 2019; HilleRisLambers et al., 2012; Mayfield & Levine, 2010). In this study, beyond the negative relationships between trait dissimilarity and spatial associations (He & Biswas, 2019), we also assessed and compared the effects of trait dissimilarity and hierarchy on pairwise spatial associations. Thereby, we were able to disentangle the relative importance of multiple assembly mechanisms in structuring co‐occurrence patterns, especially the mechanisms of environmental filtering and hierarchical competition, which lead to indistinguishable co‐occurrence patterns. By linking the pairwise spatial associations, which reflect signatures left by different assembly mechanisms, to the effects of trait dissimilarity and trait hierarchy, our study provides alternative perspectives and better understanding of the underlying mechanisms that govern the co‐occurrence pattern (He & Duncan, 2000; Wiegand et al., 2007; Wiegand & Moloney, 2014).

However, the stronger effects of trait hierarchy than trait dissimilarity were found largely consistent for both sapling and adult trees for most individual traits (LA, SLA, WD, and WDMC) and the integrated trait (Figures 4 and 5, Figures S1–S8), reflecting the consistent assembly mechanisms in structuring the co‐occurrence patterns across life stages in the study forest. However, this is not without exception. For example, for *H*_max_ the trait dissimilarity effects on adult trees were consistently greater than the effects of trait hierarchy (Figures S9 and S10), which is opposite to the effects of *H*_max_ on sapling species and the effects of other traits. This exception might suggest that *H*_max_ captured the shift in assembly mechanisms from saplings to adults. For saplings, hierarchical competition outweighed environmental filtering, while for adults, the importance of environmental filtering surpassed hierarchical competition (Spasojevic et al., 2014).

The absence of limiting similarity found in both our study and He and Biswas (2019) does not necessarily suggest the absence of competition as stated in He and Biswas (2019), in fact competition played an important role in structuring the co‐occurrence in the HSD forest plot (Figures 4 and 5, Figures S1–S10). Our study supports that neighborhood competition is more likely to be driven by trait hierarchy, rather than trait dissimilarity, which is consistent with the findings of previous studies (Carmona et al., 2019; Kraft et al., 2014; Kunstler et al., 2012). If trait dissimilarity was positively related to pairwise spatial associations, we would infer that trait dissimilarity affects the neighborhood competition and that dissimilar species were more likely to co‐occur. However, this is not the case in this study and we instead show that the effects of hierarchical competition likely exclude inferior competitors and we therefore speculate that neighborhood competition in our forest plot was more likely to be driven by trait hierarchy but not by trait dissimilarity as presumed (Carmona et al., 2019; Kraft et al., 2014; Kunstler et al., 2012). This begs the question of how species coexist in our forest, and we surmise that species are locally competitively superior, based on underlying environmental gradients and the dominant species changes with changes in environmental conditions (Cadotte & Tucker, 2017).

Although negative relationships between spatial associations and trait dissimilarity were generally found in this study and He and Biswas (2019), there existed some inconsistencies. For example, we detected significant negative relationships between spatial associations and trait dissimilarity of WD and WDMC for saplings, while He and Biswas (2019) failed to detect such relationships. This inconsistency might be caused by the methodological differences between our work and He and Biswas (2019). The first difference is that we used the method of standardized effect size to assess the magnitude of departure from independency of the pairwise spatial associations, while He and Biswas (2019) used the area under the pcf function. The second difference is that we used the coefficients of the linear mixed‐effects model to measure the relationships between trait dissimilarity and spatial associations, while He and Biswas (2019) used the Mantel correlation.

In this study, we observed the strength of trait dissimilarity on spatial associations decreased with the increasing spatial scales for both saplings and adult trees (Figure 3). Such spatial scale effects could be caused by the scale‐dependent nature of the relative importance of different assembly mechanisms. As the negative relationships between trait dissimilarity and spatial associations were more likely driven by neighborhood competition than environmental filtering in this study (Figures 4 and 5, Figures S1–S10), and the effects of neighborhood competition mainly operated at local scales within several meters and decayed with increasing spatial scale (Jin et al., 2020; Wang et al., 2015), it therefore came as no surprise that the integrative effects of neighborhood competition and environmental filtering decreased with spatial scales up to *r* = 50 m in this study. It is also notable that, for saplings, the magnitude of trait dissimilarity effects slightly increased with the spatial scales from *r* = 30 to *r* = 50 m (for spatial patterns assessed by pcf). This might suggest that the relative importance of environmental filtering increased at *r* > 30 m for saplings. But, this trend was not observed for adult trees (Figure 3), which suggests the scale threshold of the transition from neighborhood competition to environmental filtering for adult trees might be greater than that for saplings.

The two metrics summarizing spatial point patterns (*g_ij_*(*r*) and *D_ij_*(*r*)) that we used in this study showed no significant differences in the effects of trait dissimilarity and trait hierarchy on spatial associations for each trait (Figures 3, 4, 5). We therefore conclude that extreme heterogeneity of species distributions was not prevalent in our forest plot (Wiegand et al., 2007). Since these two summary statistics, respectively, characterize the mean number of individuals and the nearest neighbors of the second species around the focal species, the findings that these two summary statistics of spatial point patterns reveal similar trait effects suggest that the neighborhood interspecific competitive effects on the focal trees come from both the average neighbor density and the nearest neighbors of the other species at least within the scale of 50 m.

However, this study is not without limitation. First, our work is constrained by the availability of trait data. On the one hand, the species‐level trait data, instead of ontogenetic trait variation, applied in this study might cause biases for detecting ontogenetic shifts in the assembly mechanisms (Spasojevic et al., 2014), since sampling of species‐level *H*_max_ was obviously biased toward adult individuals, and sampling of other traits in this study could also be ontogenetically biased toward early stages (He et al., 2018). On the other hand, though the six traits used in this study are all of ecological importance for tree species, incorporation of other functional traits (e.g., root biomass and other belowground traits) into the analysis might capture different mechanisms than what the current traits captured (Kraft et al., 2015). However, we currently lack the data of ontogenetic trait variation and root traits to calibrate these potential biases. The second limitation could arise from species selection. We specifically selected common species in this study to ensure sufficient sample size for reliable spatial association analysis. The assembly mechanisms revealed from this study may not reflect the mechanisms regulating the co‐occurrence of rare species or between rare and common species (Chen et al., 2019; Mi et al., 2012).

## 5 CONCLUSIONS

In conclusion, we disentangled the assembly mechanisms of limiting similarity, environmental filtering, and hierarchical completion in structuring the co‐occurrence patterns in our forest community by assessing and comparing the effects of trait dissimilarity and trait hierarchy on pairwise spatial associations. More specifically, we found that limiting similarity was absent and hierarchical competition played a more important role than environmental filtering in structuring the co‐occurrence patterns for common species. It is noted that not every single species showed trait effects, suggesting the possibility that other assembly mechanisms rather than the three we tested (i.e., limiting similarity, hierarchical competition, and environmental filtering) could operate in our forest as well, for example, density‐dependent pathogen effects (Chen et al., 2019). This study also reinforced the importance of trait hierarchy, rather than trait dissimilarity, in driving interspecific competition.

## CONFLICT OF INTEREST

The authors declare no conflict of interest.

## AUTHOR CONTRIBUTIONS

**Deyi Yin:** Data curation (lead); Formal analysis (lead); Writing‐original draft (lead); Writing‐review & editing (lead). **Yu Liu:** Conceptualization (supporting); Supervision (lead). **Qing Ye:** Funding acquisition (supporting). **Marc W. Cadotte:** Writing‐review & editing (supporting). **Fangliang He:** Conceptualization (supporting); Investigation (lead); Writing‐review & editing (lead).

### OPEN RESEARCH BADGES

This article has earned an Open Data Badge for making publicly available the digitally‐shareable data necessary to reproduce the reported results. The data is available at https://doi.org/10.5061/dryad.51c59zw7k.

## Supporting information

Supplementary Material

## Data Availability

The data of species distribution of HSD plot and functional traits used in this article are archived in Dryad (https://doi.org/10.5061/dryad.51c59zw7k).
