# Supplementary material for: Trait hierarchies are stronger than trait dissimilarities in structuring spatial co‐occurrence patterns of common tree species in a subtropical forest
Source: Ecol Evol. 2021 May 1;11(12):7366–77. doi: 10.1002/ece3.7567 (PMC8216963; doi:10.1002/ece3.7567)

Supplementary Materials

Figure Legends

**Figure S1.** Comparison between the strengths of absolute and hierarchical distances of LA on spatial associations for the sapling and adult tree species. The strengths of absolute and hierarchical trait distances are respectively given by the absolute values of the coefficients of the variables of hierarchical and absolute trait distances of different functional traits in the model of Eq (2). Histograms, distributions and mean values of absolute values of the coefficient estimated for each focal species are plotted (blue for absolute trait distance and orange for hierarchical trait distance). ﻿The conditional R squared (R_c_^2^ ) for each model and p-value for the paired t-test for the strengths of absolute and hierarchical trait distances for each focal species are reported in each panel, where *** indicates p < 0.001, ** indicates p < 0.01, * indicates p <0.05, . indicates p < 0.1, ns indicates p > 0.1. The results presented here are for spatial associations assessed by bivariate pair-correlation function (*g_ij_*(*r*), pcf) at *r*=5 m, 30 m, and 50 m.

**Figure S2.** Similar to Figure S2, but the results are for the comparison between the strengths of absolute and hierarchical distances of LA on spatial associations assessed by bivariate distribution function of nearest neighbor (*D_ij_*(*r*), D function), at *r*=5 m, 30 m, and 50 m.

**Figure S3.** Similar to Figure S2, but the results are for the comparison between the strengths of absolute and hierarchical distances of SLA on spatial associations assessed by bivariate pair-correlation function (*g_ij_*(*r*), pcf), at *r*=5 m, 30 m, and 50 m.

**Figure S4.** Similar to Figure S2, but the results are for the comparison between the strengths of absolute and hierarchical distances of SLA on spatial associations assessed by bivariate distribution function of nearest neighbor (*D_ij_*(*r*), D function), at *r*=5 m, 30 m, and 50 m.

**Figure S5.** Similar to Figure S2, but the results are for the comparison between the strengths of absolute and hierarchical distances of WD on spatial associations assessed by bivariate pair-correlation function (*g_ij_*(*r*), pcf), at *r*=5 m, 30 m, and 50 m.

**Figure S6.** Similar to Figure S2, but the results are for the comparison between the strengths of absolute and hierarchical distances of WD on spatial associations assessed by bivariate distribution function of nearest neighbor (*D_ij_*(*r*) , D function), at *r*=5 m, 30 m, and 50 m.

**Figure S7.** Similar to Figure S2, but the results are for the comparison between the strengths of absolute and hierarchical distances of WDMC on spatial associations assessed by bivariate pair-correlation function (*g_ij_*(*r*), pcf), at *r*=5 m, 30 m, and 50 m.

**Figure S8.** Similar to Figure S2, but the results are for the comparison between the strengths of absolute and hierarchical distances of WDMC on spatial associations assessed by bivariate distribution function of nearest neighbor (*D_ij_*(*r*) , D function), at *r*=5 m, 30 m, and 50 m.

**Figure S9.** Similar to Figure S2, but the results are for the comparison between the strengths of absolute and hierarchical distances of H_max_ on spatial associations assessed by bivariate pair-correlation function (*g_ij_*(*r*), pcf), at *r*=5 m, 30 m, and 50 m.

**Figure S10.** Similar to Figure S2, but the results are for the comparison between the strengths of absolute and hierarchical distances of H_max_ on spatial associations assessed by bivariate distribution function of nearest neighbor (*D_ij_*(*r*) , D function), at *r*=5 m, 30 m, and 50 m.

Figure S1.


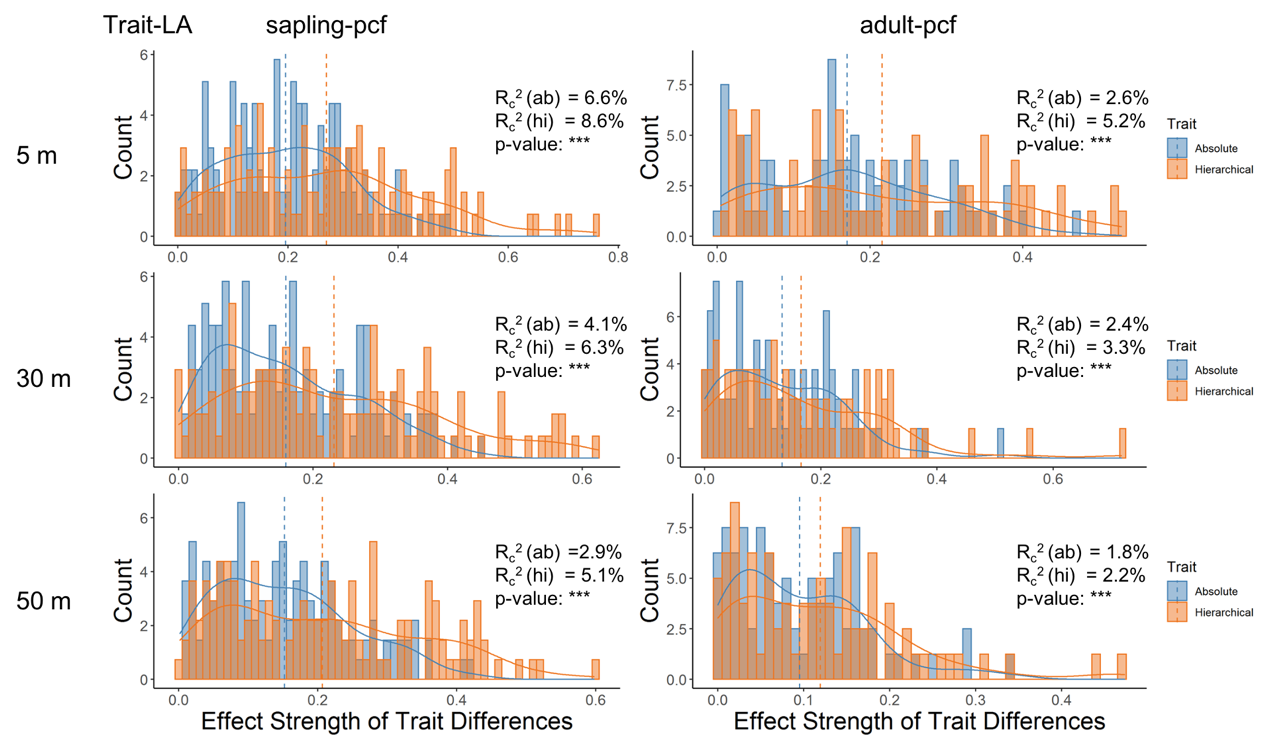


Figure S2.


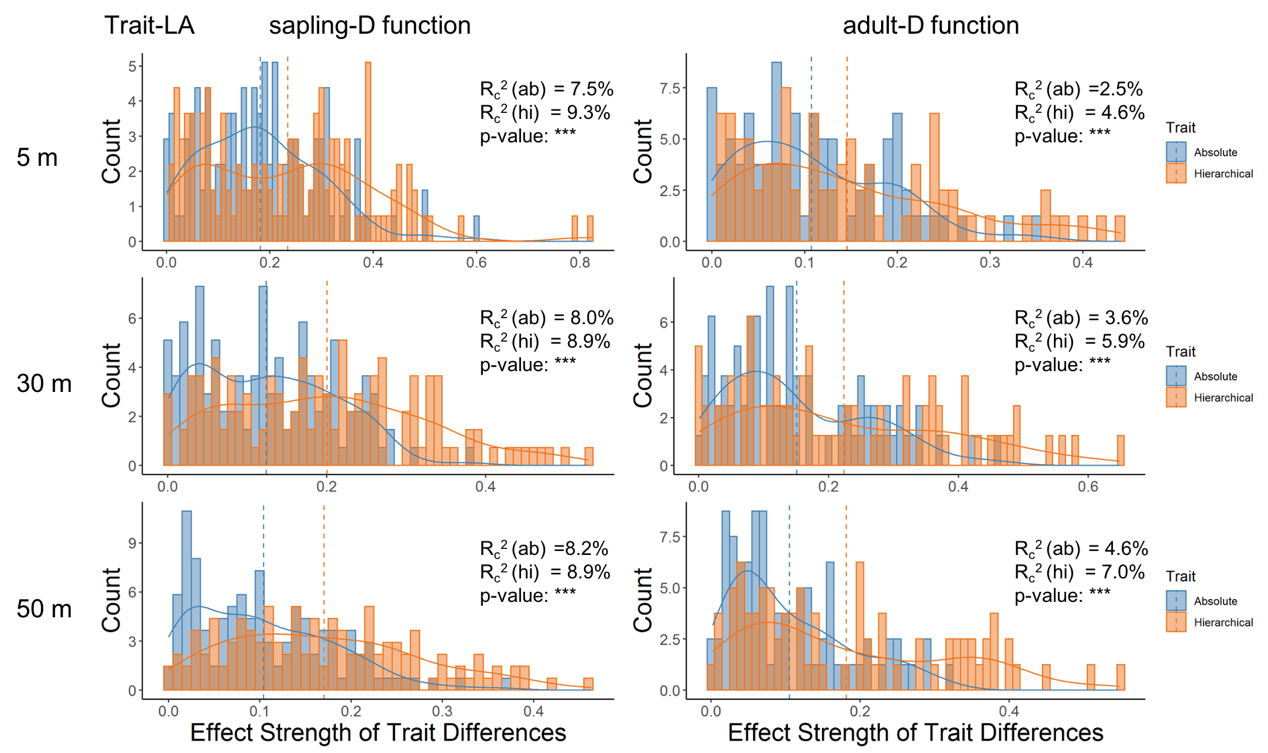


Figure S3.


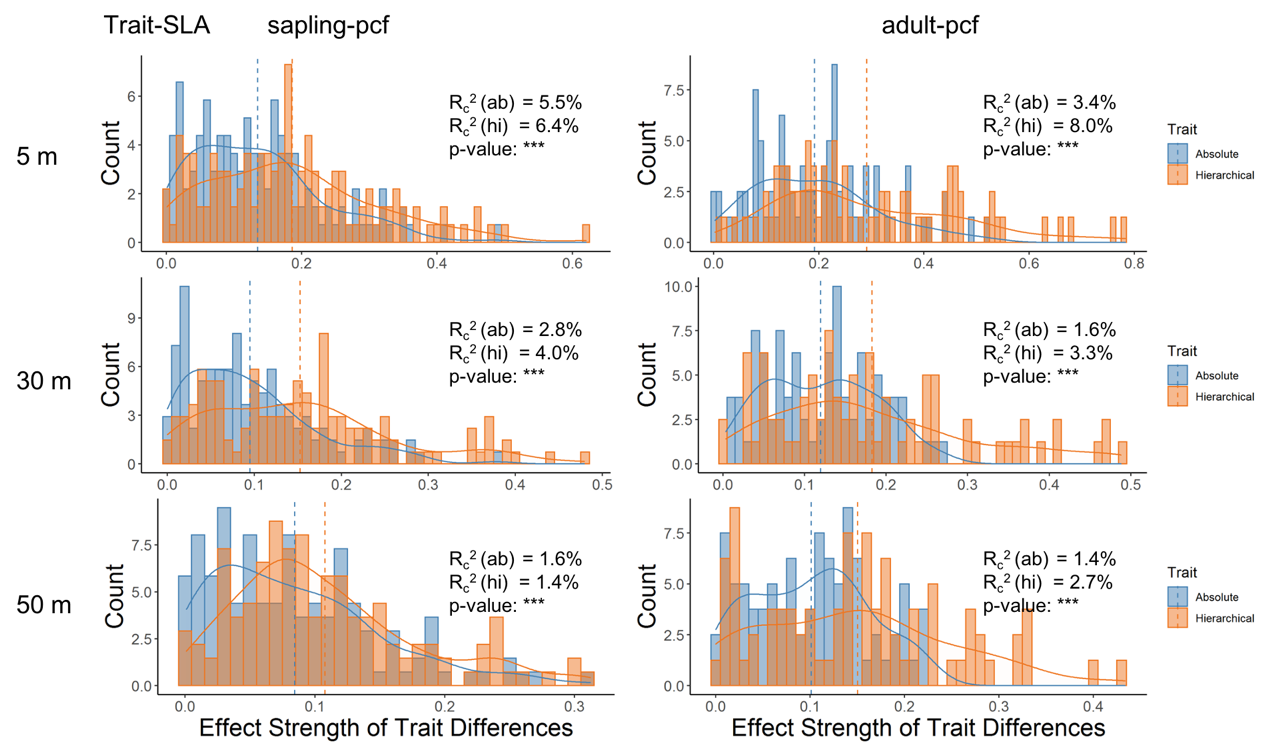


Figure S4.


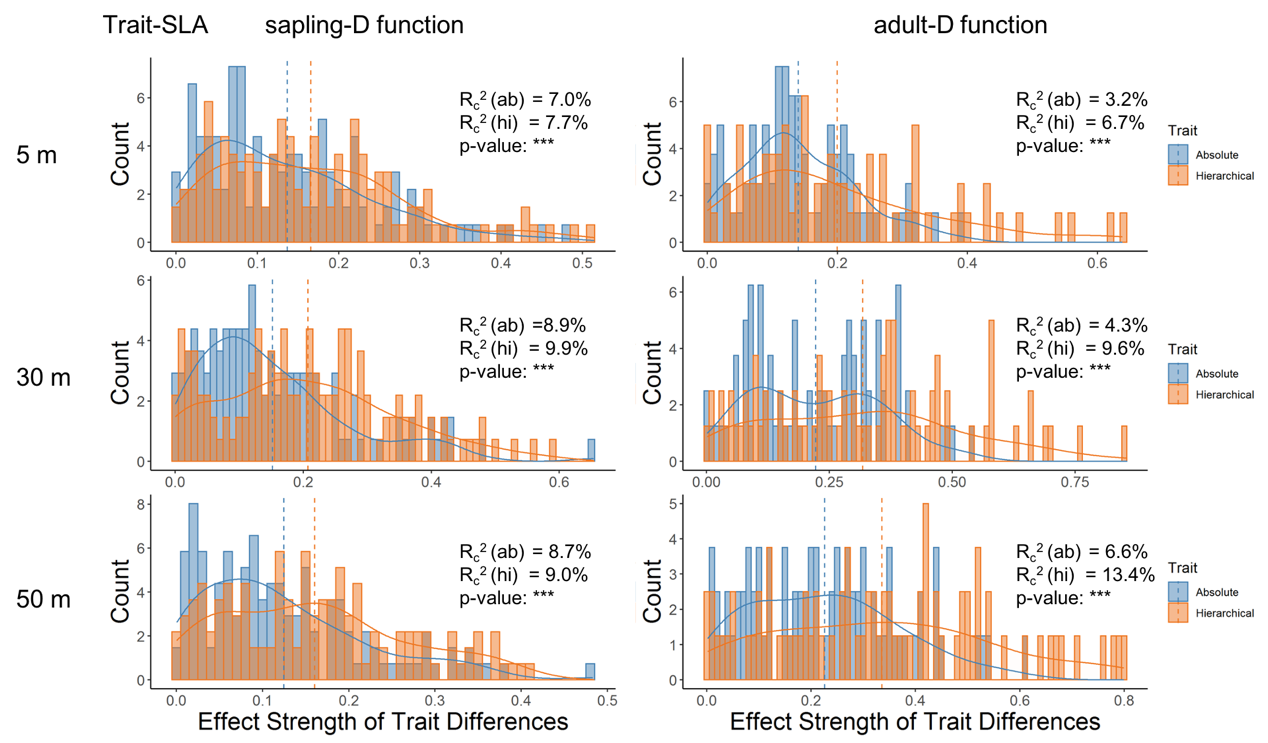


Figure S5.


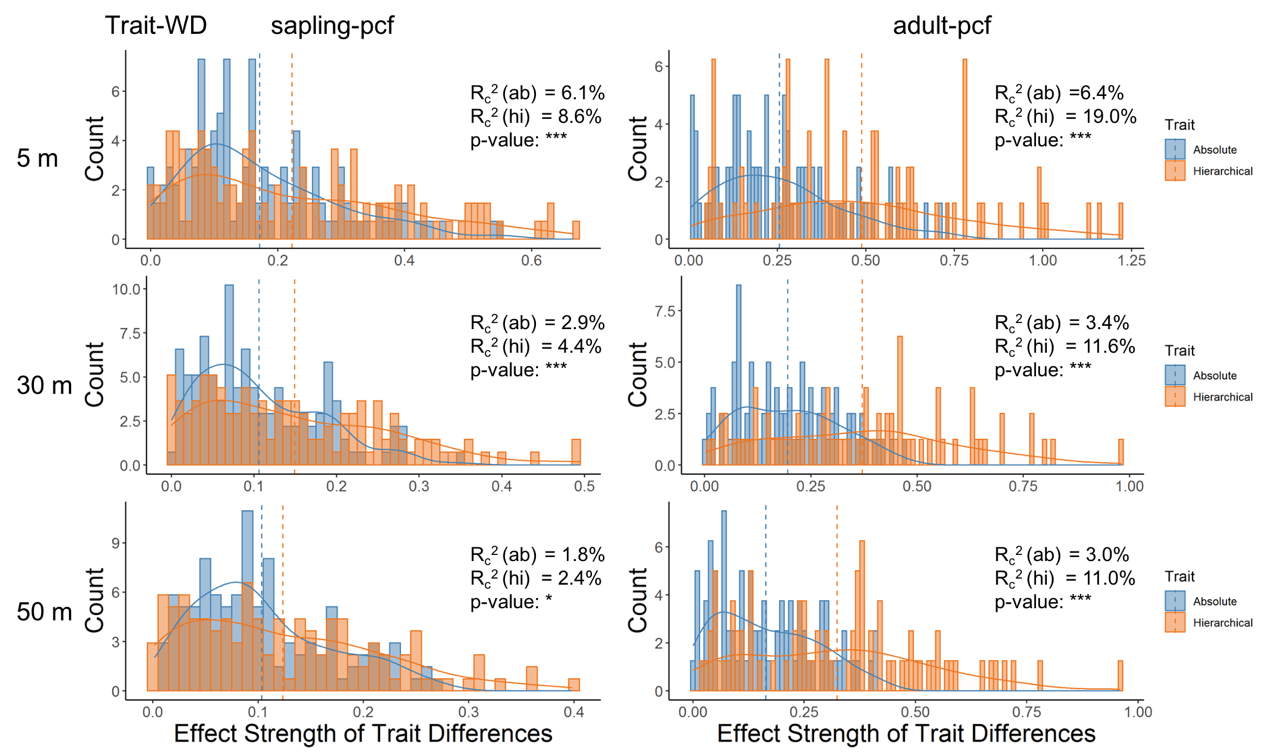


Figure S6.


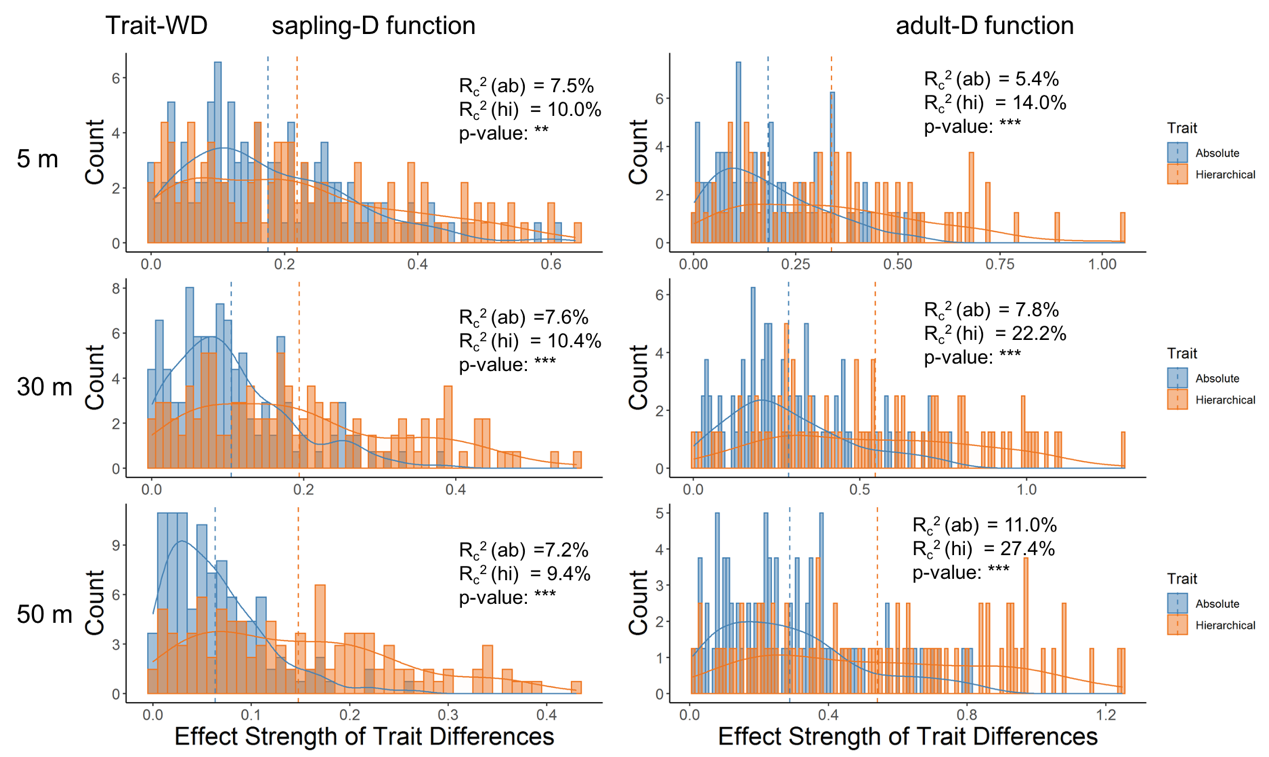


Figure S7.


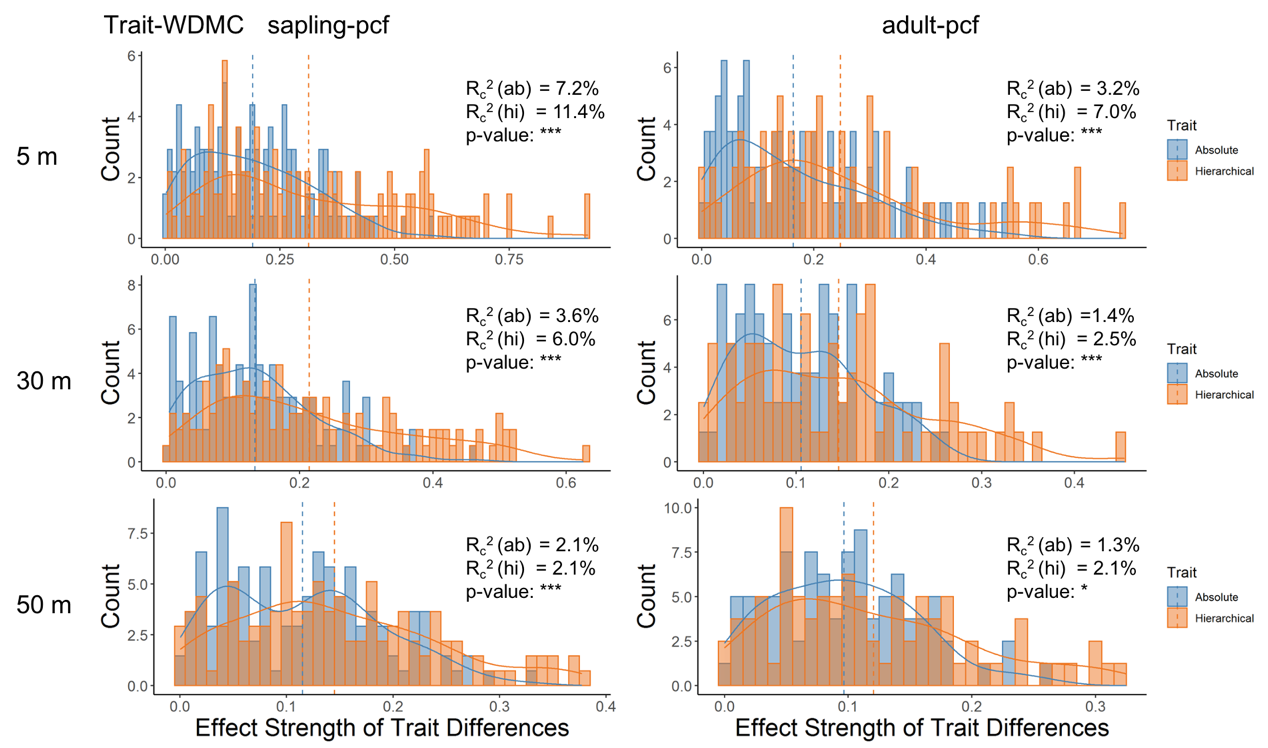


Figure S8.


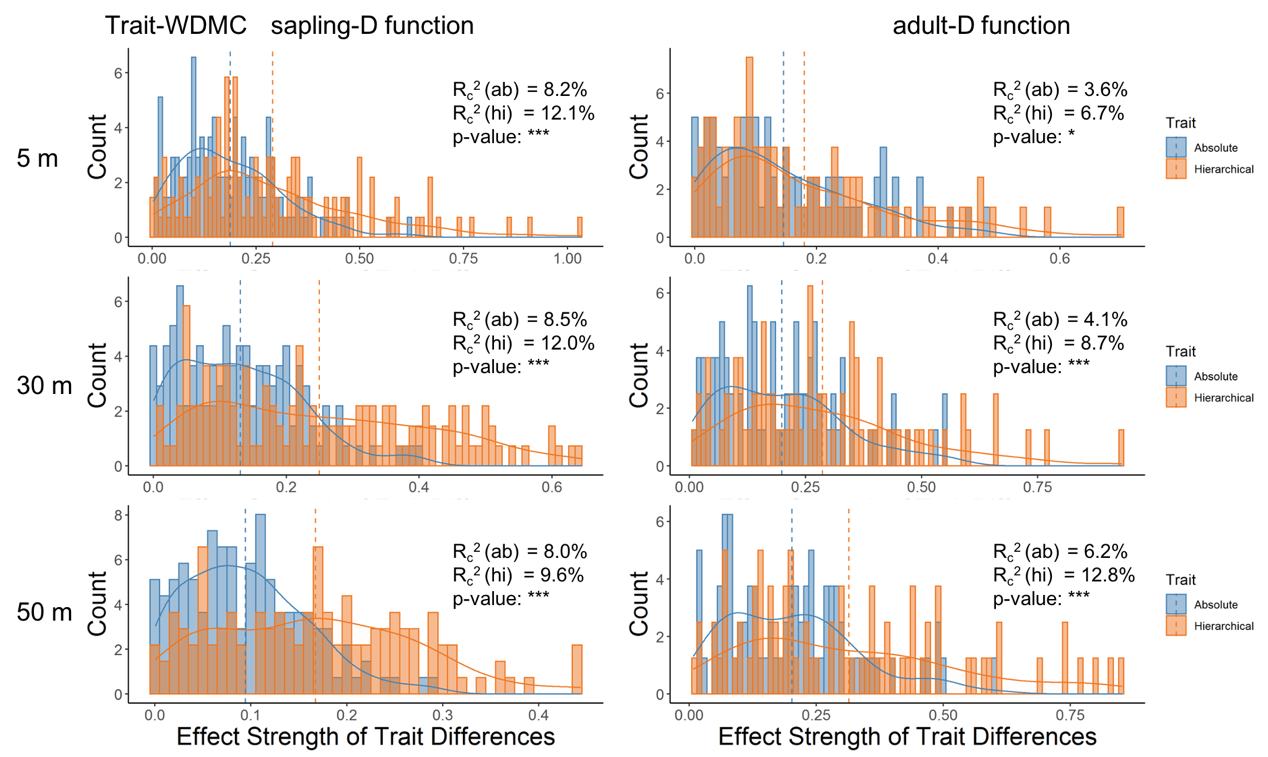


Figure S9.


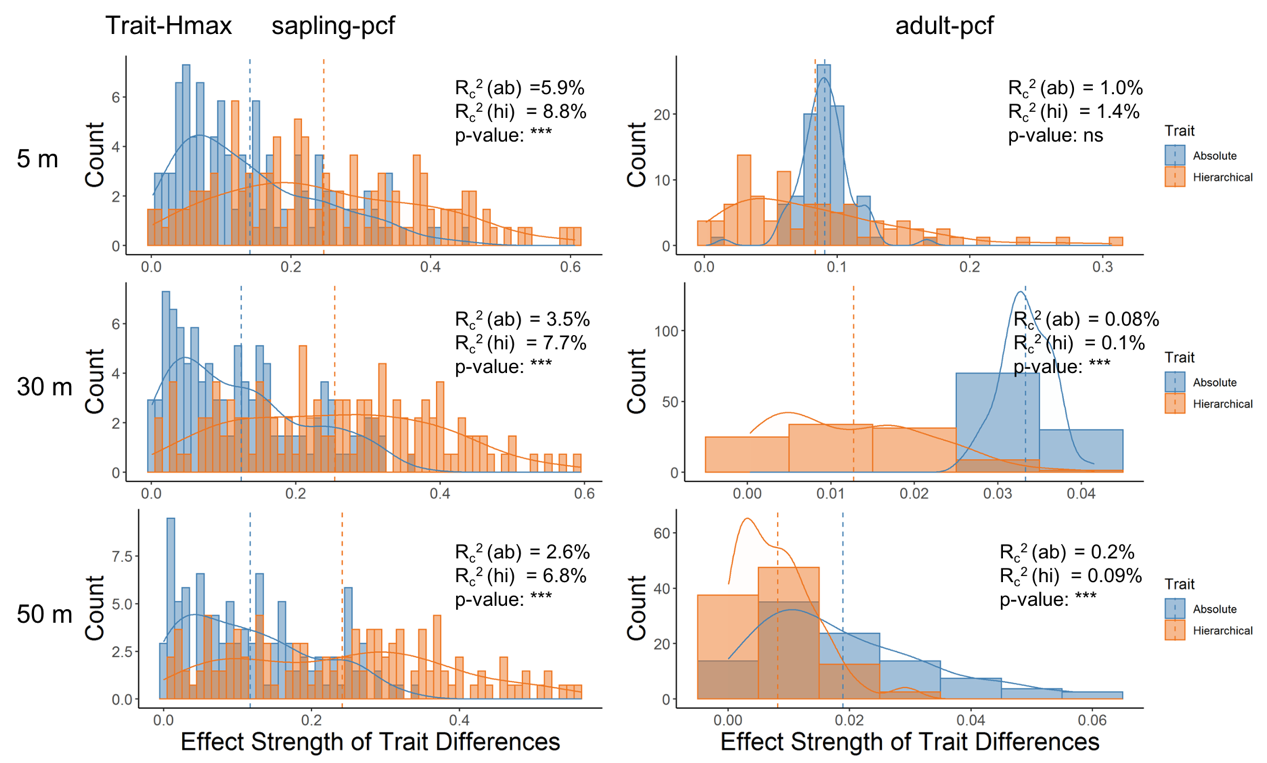


Figure S10.


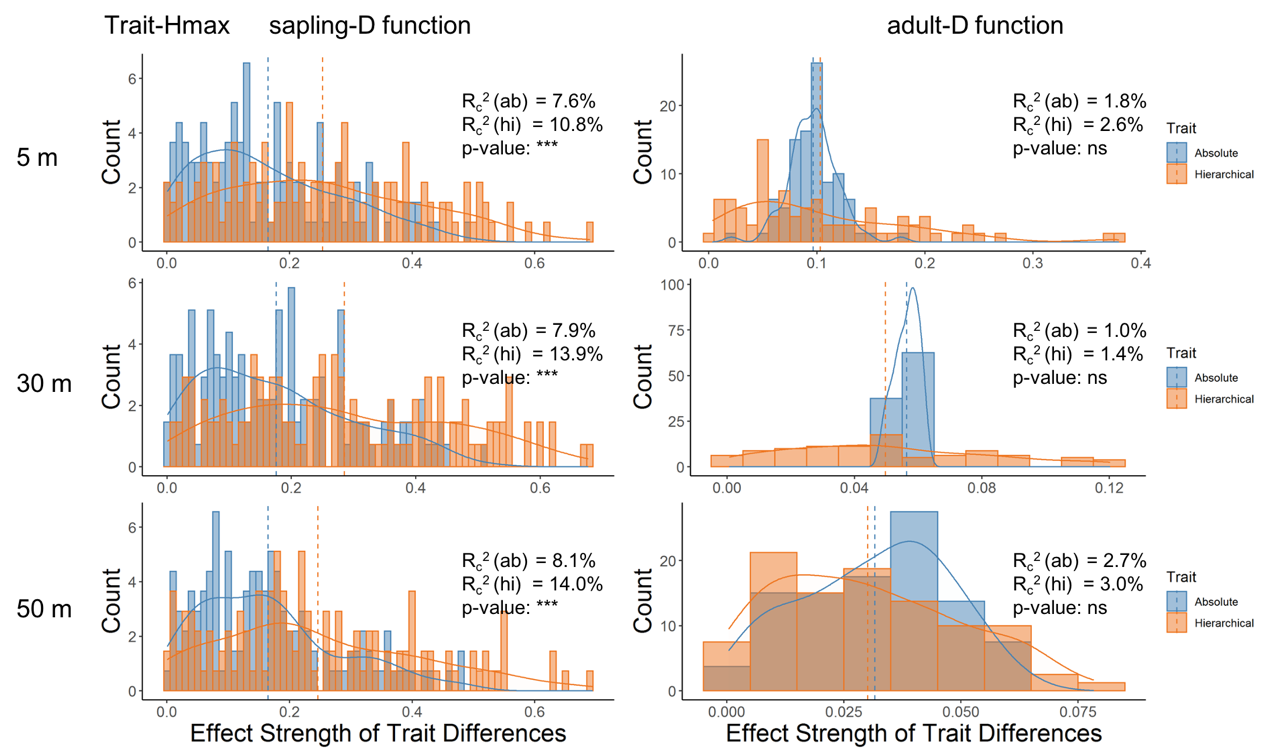

Supplement: Supplementary file 1 — Supplementary Material [file ECE3-11-7366-s001.docx]
